# Metabolomics Based Profiling of Dexamethasone Side Effects in Rats

**DOI:** 10.3389/fphar.2018.00046

**Published:** 2018-02-16

**Authors:** Abeer K. Malkawi, Karem H. Alzoubi, Minnie Jacob, Goran Matic, Asmaa Ali, Achraf Al Faraj, Falah Almuhanna, Majed Dasouki, Anas M. Abdel Rahman

**Affiliations:** ^1^Department of Clinical Pharmacy, Faculty of Pharmacy, Jordan University of Science and Technology, Irbid, Jordan; ^2^Department of Comparative Medicine, King Faisal Specialist Hospital and Research Center, Riyadh, Saudi Arabia; ^3^Department of Genetics, King Faisal Specialist Hospital and Research Center, Riyadh, Saudi Arabia; ^4^Molecular and Cell Biology, College of Public Health, Medical and Veterinary Sciences, James Cook University, Townsville, QLD, Australia; ^5^Department of Radiologic Sciences, Faculty of Health Sciences, American University of Science and Technology, Beirut, Lebanon; ^6^College of Medicine, Alfaisal University, Riyadh, Saudi Arabia; ^7^Department of Chemistry, Memorial University of Newfoundland, St. John’s, NL, Canada

**Keywords:** pharmacometabolomics, dexamethasone, glucocorticoids, mass spectrometry, metabolomics, osteoporosis, rats, side-effects

## Abstract

Dexamethasone (Dex) is a synthetic glucocorticoid that has anti-inflammatory and immunosuppressant effects and is used in several conditions such as asthma and severe allergy. Patients receiving Dex, either at a high dose or for a long time, might develop several side effects such as hyperglycemia, weight change, or osteoporosis due to its *in vivo* non-selectivity. Herein, we used liquid chromatography-tandem mass spectrometry-based comprehensive targeted metabolomic profiling as well as radiographic imaging techniques to study the side effects of Dex treatment in rats. The Dex-treated rats suffered from a ∼20% reduction in weight gain, hyperglycemia (145 mg/dL), changes in serum lipids, and reduction in total serum alkaline phosphatase (ALP) (∼600 IU/L). Also, compared to controls, Dex-treated rats showed a distinctive metabolomics profile. In particular, serum amino acids metabolism showed six-fold reduction in phenylalanine, lysine, and arginine levels and upregulation of tyrosine and hydroxyproline reflecting perturbations in gluconeogenesis and protein catabolism which together lead to weight loss and abnormal bone metabolism. Sorbitol level was markedly elevated secondary to hyperglycemia and reflecting activation of the polyol metabolism pathway causing a decrease in the availability of reducing molecules (glutathione, NADPH, NAD^+^). Overexpression of succinylacetone (4,6-dioxoheptanoic acid) suggests a novel inhibitory effect of Dex on hepatic fumarylacetoacetate hydrolase. The acylcarnitines, mainly the very long chain species (C12, C14:1, C18:1) were significantly increased after Dex treatment which reflects degradation of the adipose tissue. In conclusion, long-term Dex therapy in rats is associated with a distinctive metabolic profile which correlates with its side effects. Therefore, metabolomics based profiling may predict Dex treatment-related side effects and may offer possible novel therapeutic interventions.

## Introduction

Dexamethasone (Dex) is a non-selective glucocorticoid (GC) drug that is widely used for immunological, allergic, and inflammatory diseases treatment via the activation of the nuclear glucocorticoid receptors (GRs). GRs are widely expressed in the body, and they promote the expression of several genes that regulate multiple metabolic pathways, such as inflammation, and glucose, lipid, and bone metabolism (Rafacho et al., 2014; Wu et al., 2014). Dex administration can cause several side effects, either at high doses or after long-term use. Insulin resistance and hyperglycemia, weight change, and hyperlipidemia are considered the primary adverse metabolic changes strongly associated with Dex administration (Hopkins and Leinung, 2005). Furthermore, the degree of osteoporosis and bone loss that stem from Dex depends on age, weight, gender, and the duration of drug use (Reid, 2000). In addition, steroid psychosis (e.g., mania, hallucinations, and delusions) is a typical irreversible central nervous system (CNS) side effect associated with the Dex treatment and other steroids (Ciriaco et al., 2013). Muscle atrophy is mediated via activation of the cellular proteolytic system (mainly the ubiquitin-proteasome system); which results in the accumulation of TRIM63 and FBXO32 in muscle, in response to Dex (Morimoto et al., 2015). These adverse effects are more likely to occur in susceptible individuals, such as pregnant women, obese subjects, or diabetic patients (van Raalte et al., 2009).

Osteoporosis is also a known side effect of long-term therapy with steroids. Multiple biomarkers useful for monitoring bone health such as related bone enzymes and bone mineral density (BMD) are commonly used in clinical practice. Osteocalcin (OC), also known as bone gamma carboxyglutamic acid protein (BGLAP) is a widely expressed protein linked to the bone formation and has an essential role in bone mineralization given its ability to bind calcium and promote osteoblast differentiation which is indicated by alkaline phosphatase (ALP) activity. However, ALP is a non-specific biomarker for bone disorders in which serum ALP level and bone density are decreased due to impaired osteogenesis (Brzoska and Moniuszko-Jakoniuk, 2004).

Pharmacometabolomic studies measure metabolites in an individual’s biological matrices to predict and evaluate the metabolism of pharmaceutical compounds and understand a drug’s pharmacokinetic profile (Jacob et al., 2017). Also, they measure within an individual the metabolic level change following a specific drug administration, to monitor its effects on individual metabolic pathways (i.e., in line with pharmacodynamics). Dex induces the metabolic changes in a complicated way involving several metabolic pathways. In a recent comprehensive metabolomic study based on a single dose exposure to Dex in healthy human volunteers, multiple metabolic changes predicted the side effects related to this exposure (Bordag et al., 2015). However, this prediction could not be validated without observing the actual side effects, which only can be done in an animal model. In this study, we evaluated the metabolomics profile (Metabotype) in an established animal model with known Dex related side effects. This holistic approach may lead to a better understanding of the mechanisms of intentional (as a nominated target of the compound) or unintentional (as side effects) responses to prolonged treatment with Dexamethasone in rats.

## Materials and Methods

### Chemicals and Materials

Sprague-Dawley (SD) rats were obtained from the Department of Comparative Medicine at King Faisal Specialist Hospital and Research Center (KFSHRC) (Riyadh, Saudi Arabia). Isoflurane for anesthesia during blood collection and sacrificing was purchased from Piramal Critical Care (Bethlehem, PA, United States). Dexamethasone phosphate, solvents, and other standard chemicals for metabolomics were obtained from Sigma–Aldrich (St. Louis, MO, United States). The reagents for the routine chemistry analyzer were purchased from Roche (Kaiseraugst, Switzerland), β-crosslaps (β–CT_x_) ELISA kit from Abbexa (Cambridge, United Kingdom), osteocalcin ELISA kit from Elabscience Biotechnology (WuHan, China).

Metabolite standards and reagents were obtained from Sigma Chemicals (St. Louis, MO, United States) at a minimum purity of 98%. Pterin (2-Amino-4-hydroxypteridine) and L-Monapterin were purchased from Schricks Laboratories (Postface, Switzerland). Isotope-labeled internal standards: 2-Amino 1,6-Hexandioc-D3, Guanosine-15N5, Inosine-15N4, D-Fructose(2-13C), Citric acid-D4, L-Citrulline-D7, Adenosine-C13, Methylmalonic acid-D3, 2-Deoxyadenosine-C13, Glucose–D7 were purchased from Cambridge Isotope, Inc. (Woburn, MA, United States). Whereas Alanine-D4, Arginine-D7, Aspartic acid-D3, Citrulline-D2, Glutamic acid-D5, Leucine-D3, Methionine-D3, Ornithine –D6, Phenylalanine-D5, Tyrosine-D4, Valine-D8, C0-Carnitine-D9, C2-Carnitine-D3, C3-Carnitine-D3, C4-Carnitine-D3, C5-Carnitine D9, C6-Carnitine-D3, C8-Carnitine-D3, C10- Carnitine-D3, C12- Carnitine-D3, C14- Carnitine-D3, C16- Carnitine-D3, C18- Carnitine-D3 were purchased from ChromoSystems (Grafelfing, Germany). All organic solvents and water used in sample and mobile phase preparations were LC-MS/MS grade and obtained from Fisher Scientific (Fair Lawn, NJ, United States).

### Animal Model

The animal study was carried out according to a protocol approved by the animal ethics committee at KFSHRC (approval number 2150016). To eliminate the potential effect of female sex hormones on bone metabolism, two groups of male SD rats (Age: 6–8 weeks, weight: 200–250 g) were used in this study. All rats were kept under standard environmental conditions with regulated temperature (20–24°C), humidity (45–50%) and 12 h/12 h light/dark cycle with free access to food and water. Rats were randomly separated into two groups (*n* = 10 each). The Dex and control (Ctrl) groups were injected intramuscularly with 2.5 mg/kg twice a week for 14 weeks with Dex and normal saline, respectively to induce most of the Dex side effects as suggested by (Liu et al., 2012). The animals were housed in the Department of Comparative Medicine’s animal facility at KFSHRC, where clinical phenotype was monitored on a weekly basis such as animal’s weight using an animal scale, blood sugar using glucometer GlucoCheck^TM^. Blood samples for routine work were collected from the tail vein once every other week, while the animals were anesthetized using isoflurane 2–3% inhalation in the CO_2_ chamber. Total serum ALP and lipid profile were measured upon sample collection using routine chemistry analyzer (Reflotron^®^ Plus Analyzer) (Roche, Switzerland). LDL was calculated using Friedewald Formula (FF) [LDL (mg/dL) = TC - HDL - TG/5.0] (Friedewald et al., 1972). Blood samples were collected into heparinized tubes and immediately centrifuged at 4,500 rcf for 10 min. Serum was transferred to a clean tube and stored at -80°C until the end of experiment for further analyses. The blood samples were stored properly after snap-freezing the metabolism using liquid nitrogen. Tibia and femur bones were collected after sacrificing the rats for further bone experiments.

### Bone Turnover Biomarkers Measurements

As biomarkers for bone resorption and formation, respectively, serum β-crosslaps (β-CTx) and osteocalcin (OC) levels were measured in both study groups using ELISA-based kits.

### Bone Micro-computerized Tomography (μ-CT) Scan

Rat femur was fixed in 3.7% paraformaldehyde for 24 h and analyzed by micro-CT (Skyscan, CT analyzer software, Bruker, MA, United States) to determine BMD.

### *In Vivo* Magnetic Resonance Imaging (MRI)

MRI acquisitions were performed in a free-breathing imaging protocol on a 4.7T Pharmascan 47/16 Bruker magnet interfaced with Para-Vision 5.1 software (Bruker Biospin GmbH, Rheinstetten, Germany) and operated at 300 mT/m and a slew rate of 2700 mT/m/s. A transmission and reception circularly polarized volume radio frequency coil (Bruker), with an inner diameter of 60 mm, was used for good homogeneity over the volume of interest.

Non-invasive MRI of the lung and abdominal organs (i.e., liver, spleen, and kidneys) were performed to assess whether the treatment with Dex has induced any alterations or inflammatory processes in the rats. Axial slices of 2 mm thickness were acquired using susceptibility-weighted gradient echo sequence with TR/TE = 300/3 ms, four averages, flip angle 30°, and 234 × 234 μmin-plane resolution for a total acquisition time of 5 min. Before imaging, a global shimming procedure was performed to optimize the magnetic field over the imaging volume of interest. An ultrapure water tube was positioned over the body of the rate as an external reference to normalize the MRI signal and allow measurement of contrast-to-noise ratio (CNR) variation in the different regions of interests.

For studying the bones of treated animals, sagittal slices were carefully positioned at the same location for all rats to encompass the knee and the lateral femur and tibia. High-resolution images with 1 mm thickness were acquired using a fast-spin echo (FSE) rapid acquisition with a refocused echoes (RARE) sequence with TR/TE = 2500/34 ms, RARE factor = 8, four averages, and 100 × 100 μm-plane resolution for a total acquisition time of 20 min.

### Bone Fracture Test

After sacrificing, tibia bone was weighed as wet, decreased in chloroform-methanol (2:1, v/v) for 48 h, and then dried at 120°C for 6 h. The dried bones were weighed again, and fracture forces were tested by using fracture instrument H5KS (Tinius Olsen, EUK). The instrument’s parameters were adjusted for stress range 200 MPa, 10% of displacement range, speed 10 mm/min, span 20 mm, and auto return off.

### Metabolomics Studies

The LC-MS/MS-based metabolomics analysis was performed using a recently developed and validated comprehensive targeted method in our lab (Jacob et al., 2018). For the extraction of the polar metabolome, one mL of cold extraction solvent (50% acetonitrile/50% methanol) was added to 100 μL rat serum samples containing an additional 10 μL of labeled internal standards. The mixture was shaken at 1000 rpm for one h at 4°C in a ThermoMixer (Eppendorf, Germany) (Abdel Rahman et al., 2014). Following extraction, samples were spun down at 14,000 rpm for 10 min at 4°C, and the supernatant was transferred to fresh tubes to be evaporated to dryness (∼1 h) in a Savant SpeedVac concentrator (Thermo Fisher Scientific Inc., CA). The dry extract was reconstituted in a mixture consisting of the mobile phase and placed in the autosamplar at 4°C for analysis. In this LC-MS/MS method, we used an Acquity UPLC-XEVO TQD tandem mass spectrometer (Waters Corporation, United States). Analytes were separated by reversed phase chromatography using Acquity UPLC C18, 1.7 μm, 2.1 mm × 100 mm column (at ambient temperature). Each sample was analyzed twice; in positive and negative ionization modes. In positive mode analysis, the mobile phase consisted of (A) 0.1% acetic acid and (B) 50% acetonitrile (ACN) and 50% Methanol (MeOH). The mobile phase, for the positive mode, was ramping from 2 to 95% for 10 min, held for 1 min at 95% then mobile phase A was ramped back to 2%, to regenerate the column for the next run. In negative mode, the mobile phase was composed of (A) 0.1% tributylamine (TBA), 0.03% acetic acid, 10% MeOH and (B) 100% ACN. Subsequently, mobile phase for negative mode was ramping from 5 to 70% for 13 min, held for 1 min at 70% and then the mobile phase A ramped back to 5%, to regenerate the column for the next run. The samples were run in the positive mode first and then run on the negative mode with an intermediate automated washing step to avoid any sample carryover. The total run time for each sample in each mode was 15 min at a flow rate of 0.3 mL/min. The samples were stored in the autosampler at 4°C, and the injection volume was 10 μl.

The targeted compounds which were prepared in 50% methanol (400 μM) were infused into XEVO TQD (Waters Corporation, United States) for optimization. The source and desolvation temperatures were set at 150°C and 250°C, respectively, while the desolvation gas was set at 500 L/h to tune molecules in both (positive and negative) polarity modes. The specific tuning parameters, such as ionization polarity, precursor and product ions, cone voltage and collision energy (CE) were obtained for each analyte. The eluted metabolites were analyzed under the optimal MS conditions listed in **Supplementary Table S1** using electrospray ionization. The cone voltage ranged from 18 to 170 V, and the collision energy ranged from 7 to 65 eV. Common MS parameters were the same as the tuning conditions described above except the desolvation temperature and gas flow which were 500°C and 1000 L/h, respectively.

A chromatographic method was developed to accommodate the best baseline separation for the targeted metabolites within 15 min of retention time; the gradient started at injection (zero dead volume). A mixture of all of these compounds was used to prepare a wide range of calibration curve (1–1000 nM) and a set of analytical quality control (QC) samples (25, 250,750 nM). The MS was maintained using a calibration kit and protocol as recommended by the manufacturer (Waters Corporation, United States).

### Data and Statistical Analysis

The raw data was analyzed using MetaboAnalyst software version 3.0 (McGill University, Montreal, QC, Canada). Features with more than 50% missing values were removed, while others with missing values were replaced with small values (half of the minimum positive values in the original data) assumed to be above the detection limit. The data was then normalized to the equivalent internal standard’s area under the peak, and then to the sample total sum to ensure normal distribution. To adjust for the differences among the study samples, data log transformation, and Pareto scaling approaches were used to make individual features more comparable. As the vast majority of the study analytes were Gaussian distributed, unpaired two-tailed Student’s *t*-test was used to compare the differences between two study groups (treated, non-treated), where the significance levels for metabolomics data were considered at a false discovery rate (FDR) corrected *p-*value < 0.05, and values were presented as mean ± SEM. The Sample Size Calculator for designing clinical research^1^ was used for the Mean-effect size analysis which was performed along all statistical analyses.

The chemometric analysis was carried out using principal component analysis (PCA) and orthogonal partial least-squares projection to latent structure-discriminant analysis (orthPLS-DA) (Xia and Wishart, 2016). OPLS-DA is a supervised multiple regression analysis for identifying the discrimination between different datasets. The bar-graphs were generated using Graph Pad Prism V. 6 (Xia et al., 2009, 2012). The FDR-corrected *p*-values are represented on the figures as 0.0001 (^∗∗∗^), 0.001 (^∗∗^), and 0.05 (^∗^). The statistically significant features between the study groups were used for pathway analysis and molecular mapping. Metabolic enrichment and pathway analyses were based on MetaboAnalyst^2^. The *Rattus norvegicus* pathway library was used. Cytoscape 3.4.0 on MetScape^3^ was used for large-scale network analysis and the visualization of the integrated metabolism pathways (Shannon et al., 2003).

## Results

### Phenotypic Changes Associated with Dex Treatment

**FIGURE 1:**
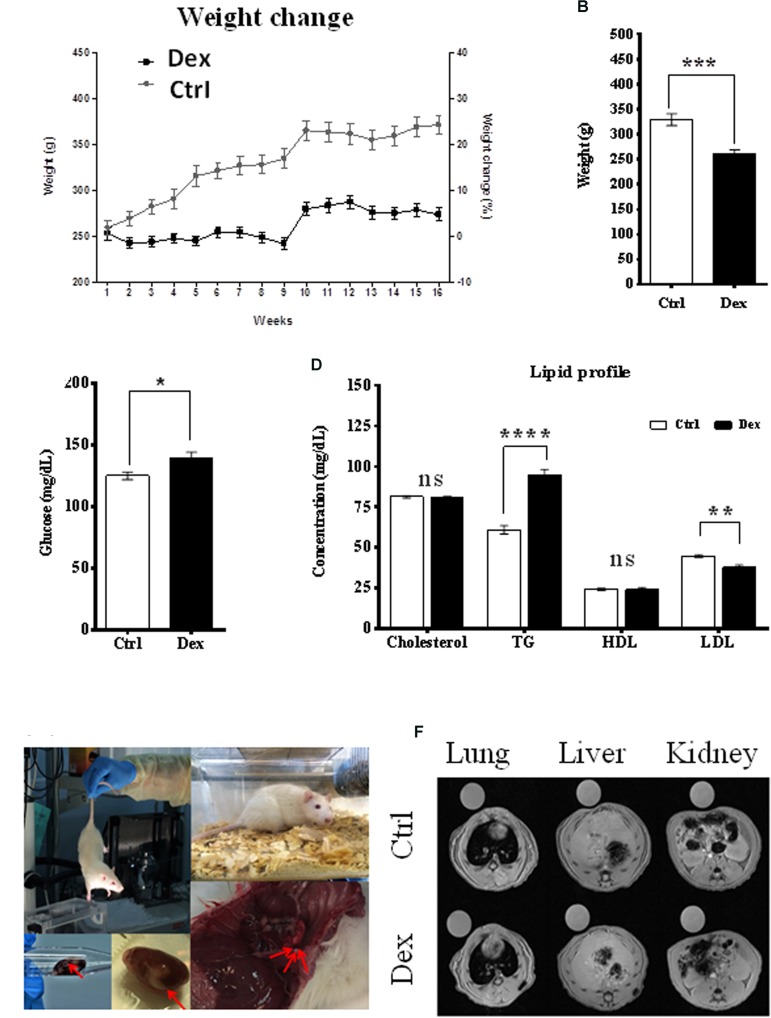
Dexamethasone effect on rats phenotypes. **(A)** The difference in weight between Ctrl and Dex treated groups during the 16 weeks period, a reduction in age-dependent increase in weight is observed in Dex treated group compared to Ctrl. **(B)** A significant reduction in total weight of Dex treated group compared to Ctrl (*p*-value 0.0002). **(C)** Significant effect of Dex treatment on blood glucose level (*p* < 0.05). **(D)** Effect of Dex on lipid profile (Cholesterol, Triglycerides (TG), High-Density Lipoprotein (HDL), Low-density lipoprotein (LDL)) in Dex treated rats compared to Ctrl. No significant effect (ns) was found on total cholesterol and HDL levels (*p* > 0.05); TG levels were significantly increased in Dex group (*p*-value 0.0001) while LDL levels were significantly decreased (*p*-value 0.001). *p*-values were calculated using unpaired student’s *t*-test, *n* = 10/group. **(E)** Changes in soft tissues in Dex treated rats. **(F)** Soft tissue MRI image (left) lung, (middle) liver, and (right) kidney and spleen in the Ctrl group and Dex treated groups.

The effects of Dex on body weight change, blood glucose level, and lipid profile were monitored during the experiment. The age-dependent body weight of the control (Ctrl) group increased as expected; however, within the Dex-treated group, it was significantly reduced by ∼20% (**Figure 1A**). Additionally, the average total weight was also significantly reduced (*p* < 0.0002) in the Dex-treated group, by 67.99 ± 14.06 g (**Figure 1B**). The blood glucose level in the Dex-treated group was substantially higher than in the Ctrl group (**Figure 1C**). In line with previous studies (Divertie et al., 1991; Plonne et al., 2001; Bruder et al., 2004), there were changes in the lipid profile of the Dex-treated rats, relative to those in the Ctrl group. The TG level changed significantly in the Dex group (*p* < 0.0001), and was the most-affected parameter in the profile; meanwhile, total cholesterol and HDL remained almost unchanged in the Dex-treated group, compared to the Ctrl group. On the other hand, LDL was markedly reduced in the Dex-treated group relative to the Ctrl group (*p* < 0.001) (**Figure 1D**).

### Soft-Tissue MRI and Autopsy Examinations

During animal sacrifice, some soft-tissue masses and changes were observed in different organs, mainly in the Dex-treated group. These included grossly hemorrhagic lung and liver, and kidney cysts as well as smaller organs than those in the Ctrl group. MRI was the technique of choice in exploring these observations in the lung, liver, kidney, spleen, and bone tissues, in both the Dex-treated and Ctrl groups. MRI images of the lung, liver, kidney and spleen tissues showed no changes between the two groups, and there was no radiographic evidence of inflammation or masses within these organs (**Figures 1E,F**).

### Bone Turnover Related Side Effects

**FIGURE 2:**
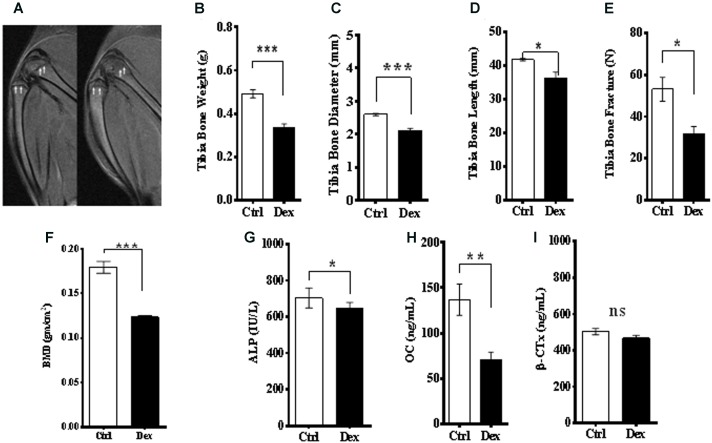
Effects of Dex treatment on bone tissues. A representative MRI of knee and lateral femur and tibia in Ctrl (left), and Dex (right) shows a bone deformity in Dex treated animals compared to Ctrl **(A)**. There was a significant reduction in dry bone weight of tibia **(B)**, tibia diameter **(C)**, tibia length **(D)** and (femur) bone mineral density measured by μ-CT **(E)**, where the *p*-values were <0.0005, <0.002, <0.05, and <0.0001, respectively. Tibia bone fracture force was significantly reduced in Dex group (*p* < 0.02) compared to Ctrl **(F)**. Serum levels of alkaline phosphatase (ALP) **(G)** and osteocalcin (OC) **(H)** are significantly reduced in Dex treated animals (*p* < 0.02 and <0.002 respectively) while β-crosslaps (β-CTx) levels were not (*p* > 0.2) **(I)**.

The effects of Dex treatment on bone tissue and on bone turnover were evaluated, using a variety of physical, chemical, and biochemical approaches. MRI of the knee, lateral femur and lateral tibia showed a deformity and a change in the structure and borders of the area (**Figure 2A**). The tibia dry-weight was measured and found to be reduced significantly in the Dex-treated group (*P* < 0.0005) compared to Ctrl (**Figure 2B**). These results were concordant with the femur weights (data are not shown). Also, there was a significant reduction in tibia-diameter (*p* < 0.0017) and length (*p* < 0.0448) due to Dex treatment (**Figures 2C,D**). To determine the bone density and resulting osteoporosis, BMD was measured *ex vivo* in tibia using μ-CT scan. Where a significant reduction in BMD (*p* < 0.0039) was identified in the Dex group compared to Ctrl (**Figure 2E**). These results are in agreement with the fracture force test that was performed on pre-treated dried tibias using H5KS (Tinius Olsen, EUK), where the fracture force required to break up the tibia was significantly lower (*p* < 0.0187) in Dex group compared to Ctrl as shown in **Figure 2F**. In Ctrls, the fracture force ranged from 50 to 60 N compared to an average of 30 N in the Dex group indicating greater than 50% reduction.

Bone metabolism has two arms; bone formation (osteogenesis) and resorption. Several biomarkers were used to evaluate the role of Dex in both processes. Serum levels of ALP and OC, as bone formation biomarkers, were reduced significantly in rats treated with Dex (*p* < 0.0115, and *p* < 0.0019, respectively) as shown in **Figures 2G,H**. The average of OC levels in Ctrl and Dex groups were found to be 140.0 ng/ml and 60.0 ng/ml, respectively, which is equivalent to more than 50% reduction. Serum levels of β-crosslaps (β–CTx), a bone resorption biomarker used clinically and in therapeutic monitoring were not changed significantly between the two groups (*p* < 0.1323) as shown in **Figure 2I**. This suggests that Dex selectively regulates (in an unknown mechanism) osteogenesis rather than bone resorption.

### Serum Metabolomics Abnormalities in Dexamethasone Treated Rats

A library of 225 targeted and clinically relevant metabolites was obtained from commercial sources and used to optimize the LC-MS/MS instrument in multiple reactions monitoring (MRM) mode. The compound specific chromatographic and mass spectrometric parameters such as retention time (RT), precursor ion (Q1), product ion (Q2), collision energy (CE) and cone voltage are summarized in **Supplementary Table S1**. This metabolomics method was validated following internationally accepted guidelines.

The metabolites area under the peak was normalized to the equivalent internal standard’s area under the peak, and the area ratio was normalized to the sample total sum to make sure all detected metabolites are within the normal distribution. The data scale was log-transformed, and the overall distribution scaled by Pareto-Scaling (mean-centered and divided by the square root of the standard deviation of each variable) showed the intensities of most of the features within the normal Gaussian distribution. Box plot analysis displayed only 50 features due to space limitation while density plot depends on all samples **Supplementary Figure S1**.

**FIGURE 3:**
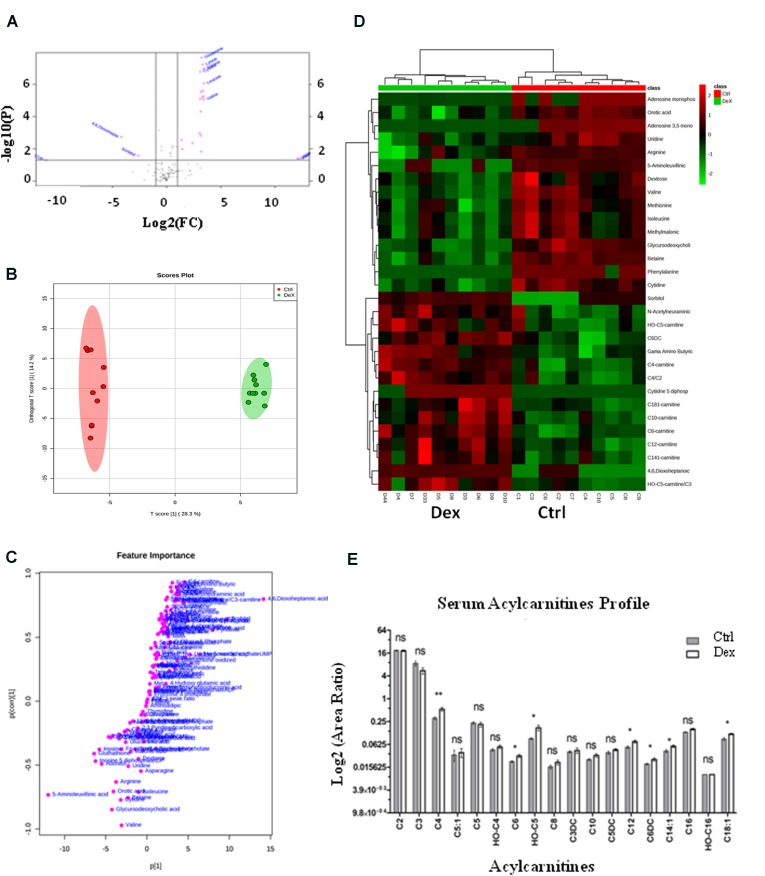
Dexamethasone effects on serum targeted metabolites. **(A)** Important features selected by volcano plot analysis with fold change threshold (*x*-axis) at 1.2 and *t*-tests threshold (*y*-axis) at 0.05. Pink circles represent features above the threshold. Please note both *p*-values and fold changes are log_10_ and log_2_ transformed, respectively. The further the feature’s position is away from the origin point (0,0), the more significant it is. **(B)** Separation of Ctrl and Dex groups using OPLS-DA score plot analysis (*Q*^2^ = 0.959). The t[1] and [1] values represent the score of each sample in principal components 1 and 2, respectively. **(C)** Loading S-plot generated by OPLS-DA analysis. The *x*-axis represents a measure of the relative abundance of ions, while the y-axis shows the correlation of each ion to the model. **(D)** Heatmap analysis showing the top significantly enriched features from *t*-test in Ctrl (red) and Dex treated (green) groups. **(E)** Serum levels of several acylcarnitines (C18:1, C14:1, C6DC, C12, OH-C5, C6, C4) were significantly increased (*p* < 0.005) in the Dex treated animals.

The detected metabolites visualized in the volcano plot (**Figure 3A**) were evaluated using *t*-test FDR-corrected *p*-value (y-axis) and fold change (FC) (*y*-axis) analyses. The significant features in the volcano plot (**Figure 3A**), are the ones that pass FC and FDR-corrected *p*-value thresholds, 1.2 and 0.05, respectively. Features shown in pink dots in the upper right and left corners of the plot, respectively represent significantly down-regulated or up-regulated metabolites upon Dex treatment. Glutamine, lysine, alanine, cysteine, leucine, 4,6, Dioxoheptanoic acid (succinylacetone), sorbitol, cAMP, CDP, valine, and a few others represent the most important features that were significantly changed (*p* < 0.05) (**Figure 3A** and **Supplementary Table S2**).

The orthogonal partial least-squares projection to latent structure-discriminant analysis (orthPLSDA), unsupervised multivariate analysis, shows the separation between Ctrl and Dex groups with *Q*^2^ = 0.959. The spacing between the two groups represents the degree of biochemical perturbation that occurred in Dex group compared to Ctrl (**Figure 3B**). The corresponding loading plot (**Figure 3C**) is used to identify biomarkers, where the features furthest from the origin are changed in their level significantly between the two groups, and they may be considered potential biomarkers of different side effects of Dex treatment.

**FIGURE 4:**
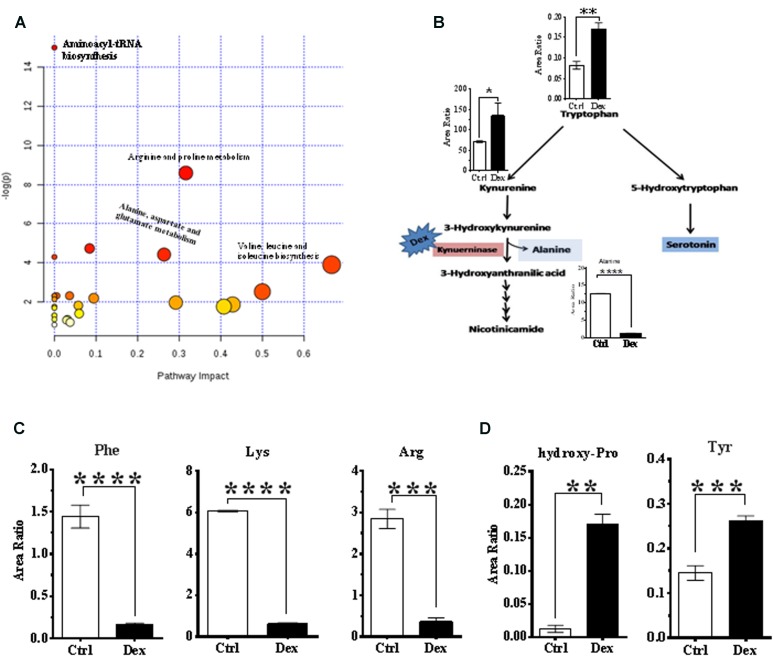
Analysis of metabolic pathways. **(A)** Summary of pathway analysis for the most relevant pathways involved under experiment conditions. **(B)** Tryptophan-kynurenine metabolism pathway. Dexamthesaone inhibits kenureninase which causes accumulation of kynurenine, 3-hydroxy-kenurenine, tryptophan and secondarily serotonin (5-hydroxytryptamine). Dex treatment causes significant elevation in serum levels of tyrosine **(C)** and hydroxyproline **(D)** and significant reduction in phenylalanine, lysine and arginine.

The most significant features, based on *t*-test, are visualized on a heatmap (**Figure 3D**), where the metabolites were segmented and clustered hierarchically based on the changing similarity across the study groups. The amino acids profile showed multiple abnormalities suggesting differential effects for Dex exposure. Several serum amino acids were down-regulated (*p* < 0.0001) in contrast to hydroxyproline, tyrosine, and tryptophan levels which were significantly increased as shown in **Figures 4C,D**. Asparagine, cystathionine, homocysteine, and 3-methylhistidine were not significantly altered under the study condition.

### Metabolomics Pathway Analysis

Pathway analysis was performed on the featured metabolites to identify the most relevant pathways involved in Dex administration. Amino acyl-tRNA biosynthesis is most significantly altered in this study which is a reflection of widespread perturbations in amino acids metabolism. Arginine and proline metabolism, pyrimidine metabolism, alanine, aspartate and glutamate metabolism, nitrogen metabolism, branched chain amino acids (valine, leucine, and isoleucine) biosynthesis were the most significantly enriched pathways (*P* < 0.05) with different impacts as shown in **Figure 4A**.

Conversely, tryptophan and kynurenine levels were increased, in Dex group, where these metabolites are involved in bone turnover processes (Yadav et al., 2008; Liu et al., 2012). Tryptophan is partially catabolized through the kynurenine pathway which produces alanine as a byproduct (**Figure 4B**). In this study, levels of phenylalanine and tyrosine were decreased, and increased, respectively in the Dex-treated group (**Figure 4C**), both of which are implicated in osteoporosis (Wu et al., 2015). Hydroxyproline is another amino acid product of collagen breakdown, and it might be considered as a bone resorption biomarker, which was markedly elevated as shown in **Figure 4D**.

## Discussion

Dexamethasone is one of the GC drugs that has many potentially serious adverse effects when used in large doses or long-term. (Harris et al., 2015) Selecting the right model for studying the drug side effect is very critical, as many factors could affect the side effects profile such as age, gender, race, pregnancy, breastfeeding, alcohol intake and impaired renal function. In humans, both the very young and very old patients are more vulnerable to such adverse reactions than other age groups. In this study, we intended to minimize the potential impact of these factors by using the male SD rat model that has been used previously to study the Dex related side effects. The clinical phenotypic and metabolic changes (metapotypes) associated with Dex were mapped in rats, where the effect size analysis showed that this model has 80% power to detect an effect size of 1.325. Rearrangement of fat deposition, known clinically as a buffalo hump and moon face, muscle atrophy and high water retention increase the body weight in humans treated with Dex or steroids in general (Chong et al., 1994; Cheskin et al., 1999; Brotman et al., 2005; Cristancho and Lazar, 2011). Herein, different from humans, age-related weight gain was observed to be severely reduced in rats treated with Dex, due to a reduction in food intake, appetite, and energy. These effects were probably mediated by the altered expression of specific genes that are involved in the synthesis of adipose tissues such as (Ob) gene (De Vos et al., 1995; Askari et al., 2005; Morimoto et al., 2015; Wu et al., 2017). Muscle atrophy is another factor that contributes to weight gain reduction due to inhibition of protein synthesis and induction of its degradation as reflected by the amino acid profile as shown in **Figures 4C,D**. The reduction in bone and other organs weight and size suggested a role for Dex in modulating overall growth which may lead to severe reduction in age-related development (Wu et al., 2017).

The Glu level was elevated in Dex-treated group, where fluctuations in postprandial glucose levels may ultimately lead to diabetes mellitus as a side effect of Dex after long-term use (Hong et al., 2014). Dex is known to play an inhibitory role in insulin signaling in different tissues (Ferris and Kahn, 2012; Wei et al., 2014), where the expression of insulin receptor (IR) is affected due to changes in protein tyrosine kinase by GCs (Caratti et al., 2015). Also, a reduction in β-cells insulin production explains the development of insulin resistance (Biddinger and Kahn, 2006). Gluconeogenesis is another contributing pathway in glucose synthesis, which might be enhanced by Dex as suggested by the elevation of serum levels of several gluconeogenic amino acids and severe weight loss complicated by the hyperglycemia mediated protein degradation (Mazziotti et al., 2011; Rafacho et al., 2014). Hyperglycemia is well known to lead to sorbitol accumulation via the activation of the polyol pathway as observed in **Figures 3A,D**.

Abnormal lipid profiles and dyslipidemia are known to occur in animals and humans after Dex treatment, where the total cholesterol and TG serum levels are elevated (Bruder et al., 2004). Dex enhances the secretion and reduces the transcription of lipoprotein lipase (LPL) enzyme from liver and adipose tissue, respectively, which consequently reduces the TG metabolism and increases its level in serum (Julve et al., 1996). This lipid profile is in agreement with the level of fatty acids (FA) as shown in the acylcarnitines profile in **Figure 3E**, where several acylcarnitines C18:1, C14:1, C6DC, C12, OH-C5, C6, and C4 were found to be significantly increased in Dex group compared to Ctrl. The Dex role in catalyzing the butyrylcholinesterase (BuChE) enzyme activity supports the animal lipid profile (Lucic Vrdoljak et al., 2005). BuChE controls the choline esters hydrolysis, which is found to be decreased in plasma after Dex treatment (Lucic Vrdoljak et al., 2005). While the exact mechanism by which Dex treatment alters HDL levels is not well known, in a rat model, Dex reduced the activity of LPL and increased the activity of lecithin:cholesterol acyltransferase (LCAT), an enzyme which can convert free cholesterol to cholesteryl ester, which then enhances the production of HDL (Jansen et al., 1992). Although the effect of Dex on the lipid profile depends on many factors such as dose and duration, as well as genetic variations and environmental factors (Ross and Marais, 2014), in this study, the dose frequency and length of exposure to Dex were sufficient to maintain both total cholesterol and HDL levels within the normal range, but increase the TG and decrease the LDL levels significantly.

A balance between bone formation and resorption is an important mechanism that maintains healthy bone structure and function. Osteoporosis, a reduction in BMD, is a crucial side effect of Dex, where the therapeutic and supratherapeutic doses of Dex reduce bone formation and resorption, respectively (Caniggia et al., 1981; Laan et al., 1993; Hansen et al., 1999; Suzuki, 2015). Physical reduction in bone weight and size due to Dex treatment are likely mediated via metabolic changes in bone tissues. Several mechanisms are involved in Dex ability to change bone tissue metabolism (Liu et al., 2015). Dex suppresses the adrenal glands and reduces the sex hormones production which inhibits osteoblast activity (Xia et al., 2014). The osteoblast activity is also regulated by growth hormone and insulin-like growth factor, muscle atrophy and myopathy (Silvestrini et al., 2000). Previously, in agreement with our findings, a significant reduction in BMD, bone mineral content, and bone surface area were reported in rats injected with Dex for 12 weeks, as well as a reduction in bone-weight (Ren et al., 2015). Bone porosity and bone strength were reported to be increased and decreased respectively in an animal model after GC use (McIlwain, 1991) which supports our finding of the significant reduction in fracture force in Dex group. In addition, abnormalities on bone MRI examination are expected, where a high dose of GCs lead to thinning and perforation of trabeculae (Ito, 2014). MRI of the knee, lateral femur and tibia in Dex-treated rats showed bone deformity.

Signs of osteoporosis were physically apparent in this study as seen in the form of changes in bone weight, volume, strength, bone deformity, and BMD. Changes in bone turnover biomarkers were considered confirmatory findings of the metabolic changes in bone tissue. Changes in OC serum level correlate with bone turnover, where the reduction in serum levels indicates a loss in bone formation (Liu et al., 2012). The bone specific-ALP isozyme is another bone remodeling biomarker (Halling Linder et al., 2013). Inborn errors of metabolism such as hypophosphatasia and Wilson disease, malnutrition, zinc deficiency as well as some drugs are known causes of reduced ALP levels and impaired bone formation via inhibition of osteoblast activity (Wu et al., 2014). An elevation in β-crosslaps (β-CTx) serum level indicates bone resorption. Enhancement of Lysophosphatidylcholines (C16:0LPC, C18:0LPC, C18:1LPC and C18:2LPC), tryptophan, and phenylalanine are known to occur in serum due to Dex administration in rats (Huang et al., 2014). Elevation of LPC level due to Dex will enhance the production of reactive oxygen species (ROS) generated by osteoclasts (Cristancho and Lazar, 2011). ROS under normal conditions lead to oxidant stress which increases bone resorption, but natural antioxidant defense mechanisms will terminate this stress (Askari et al., 2005). If this is insufficient, then more bone loss and lower BMD will occur (Hong et al., 2014). In this study, the anti-oxidant glutathione was not significantly differentially expressed, which probably contributed to osteoporosis. However, the full anti-oxidants profile was not evaluated. In osteoporotic rats, elevated levels of LPC due to an imbalance between antioxidant–oxidation processes may lead to increased carbonyl content and protein oxidation (Harris et al., 2015). Several studies have shown osteoporosis metabolic changes after Dex administration, where alanine and serotonin (5-HT) are the decomposed and the hydroxylated products of tryptophan, respectively (Mohammad-Zadeh et al., 2008; Chabbi-Achengli et al., 2012; de Vernejoul et al., 2012; Michalowska et al., 2015). Alanine levels in osteoporosis have been reported to be reduced in a rat model (Almon et al., 2005). Furthermore, other studies suggested an inhibitory effect of 5-HT on bone formation with 5-HT reducing proliferation of murine primary osteoblasts and inhibiting nitric oxide release from mouse-derived osteoblasts (Ferris and Kahn, 2012). A recent study in osteoporosis rat model reported a significant increase in tryptophan levels, where the catabolism of tryptophan to alanine was interrupted, and the hydroxylation of tryptophan to serotonin was enhanced (Almon et al., 2005).

### Dexamethasone Related Metabotypes

Rat sera exposed to Dex were profiled using an LC-MS/MS-based comprehensive targeted metabolomics approach developed by our group (Abdel Rahman et al., 2013, 2014). The profile suggested a significant perturbation in several pathways such as amino acid metabolism, pyrimidine metabolism, nitrogen metabolism, and other pathways as summarized in **Table 1**.

**Table 1:** Detailed pathway analysis of significantly differentially expressed metabolites.

Pathway	Total	Hits	Raw *p*	-Log(P)	Impact
Aminoacyl-tRNA biosynthesis	67	10	3.08E-07	14.993	0
Arginine and proline metabolism	44	6	0.000187	8.5867	0.31604
Pyrimidine metabolism	41	4	0.008808	4.7321	0.08466
Alanine, aspartate and glutamate metabolism	24	3	0.012032	4.4202	0.26371
Nitrogen metabolism	9	2	0.013598	4.2979	0
Valine, leucine and isoleucine biosynthesis	11	2	0.020248	3.8997	0.66666
Phenylalanine, tyrosine and tryptophan biosynthesis	4	1	0.08029	2.5221	0.5
Glutathione metabolism	26	2	0.098895	2.3137	0.00573
Galactose metabolism	26	2	0.098895	2.3137	0.03644
D-Glutamine and D-glutamate metabolism	5	1	0.099369	2.3089	0
Biotin metabolism	5	1	0.099369	2.3089	0
Cysteine and methionine metabolism	28	2	0.11217	2.1878	0.09464
Cyanoamino acid metabolism	6	1	0.11806	2.1365	0
Glycine, serine and threonine metabolism	32	2	0.14	1.9661	0.29197
Taurine and hypotaurine metabolism	8	1	0.15434	1.8686	0.42857
Purine metabolism	68	3	0.16275	1.8156	0.05757
Aminoacyl-tRNA biosynthesis	67	10	3.08E-07	14.993	0
Arginine and proline metabolism	44	6	0.000187	8.5867	0.31604
Pyrimidine metabolism	41	4	0.008808	4.7321	0.08466
Alanine, aspartate and glutamate metabolism	24	3	0.012032	4.4202	0.26371
Nitrogen metabolism	9	2	0.013598	4.2979	0
Valine, leucine and isoleucine biosynthesis	11	2	0.020248	3.8997	0.66666
Phenylalanine, tyrosine and tryptophan biosynthesis	4	1	0.08029	2.5221	0.5
Glutathione metabolism	26	2	0.098895	2.3137	0.00573
Galactose metabolism	26	2	0.098895	2.3137	0.03644
D-Glutamine and D-glutamate metabolism	5	1	0.099369	2.3089	0
Biotin metabolism	5	1	0.099369	2.3089	0
Cysteine and methionine metabolism	28	2	0.11217	2.1878	0.09464
Cyanoamino acid metabolism	6	1	0.11806	2.1365	0
Glycine, serine and threonine metabolism	32	2	0.14	1.9661	0.29197
Taurine and hypotaurine metabolism	8	1	0.15434	1.8686	0.42857
Purine metabolism	68	3	0.16275	1.8156	0.05757


The conversion of protein to carbohydrate through gluconeogenesis is stimulated by GCs which then promotes the storage of carbohydrate as glycogen (Kaplan and Shimizu, 1963). The expression of several serum amino acids after Dex administration was low due to its mobilization from protein and its subsequent breakdown as a source of carbon during gluconeogenesis. However, these animals could not mobilize amino acids adequately, indicating that cortisol played a role in the mobilization process (Christiansen et al., 2007). This explains the severe reduction in age-dependent weight gain of the Dex group as shown mainly in the markedly down-regulated phenylalanine as a marker for overall protein breakdown (**Figure 4D**). However, the amino acid profile in this animal model was not similar to that observed in humans where multiple amino acids (Ala, Met, Asn, Phe, Pro, and Ser) were elevated after a single dose exposure to Dex, which reflected protein degradation of skeletal muscle (Bordag et al., 2015). In addition, this amino acid profile supported the phenotypes of muscle atrophy and weight loss observed in these animals. Muscle atrophy resulted from protein degradation, where Dex systemic administration upregulates glutamine synthetase, which enhances the production of the gluconeogenic amino acid glutamine by promoting protein catabolism (Falduto et al., 1989).

The regulation of insulin signaling and homeostasis are quite complex and involve multiple molecules such as fatty acid synthase (FAS) whose expression is stimulated by insulin which leads to storage of excess glucose into adipocytes as fat (Mancia, 2014). SREBF1 is known to regulate cholesterol synthesis, where its expression in adipocytes along with FAS is inhibited by leptin, a hormone which regulates food intake and fat metabolism (Considine et al., 1997; Castillo et al., 2017). Dex stimulates leptin release from adipocytes and reduces the expression of SREBPs and FAS which then result in increased serum acylcarnitines as shown in **Figure 3E** (Considine et al., 1997). Leptin also regulates body weight by decreasing food intake, increasing energy expenditure, and inhibiting fatty acid synthesis, which explains the reduction in weight gain in the Dex group as shown in **Figure 1A**. To support the role of Dex in regulating the release of leptin from adipose tissue, the serum glucose level was found to be increased significantly (*p* < 0.05) suggesting lower levels of insulin. Increased lipolysis is indicated by the abnormal post-Dex treatment serum lipids profile which showed elevated acylcarnitines (such as C18:1, C14:1, C6DC, C12, HO-C5, C6, HO-C4, and C4) which is indicative of increased free fatty acids.

Hydroxyproline, a major component of collagen and along with proline they play a key role in its stability. It is considered a urinary bone resorption biomarker which is produced via collagen breakdown (Seibel, 2005). Elevated serum hydroxyproline observed in this study reflects the effect of Dex on collagen breakdown and induction of bone resorption but not to a level sufficient to raise serum levels of β-CTx. Therefore, serum hydroxyproline was a more sensitive marker that represents bone resorption earlier than β-CTx. Coupled with a significant reduction in OC level, this combination suggested the occurrence of osteoporosis due to severe loss of bone formation with some increase in bone resorption activity.

Tryptophan is partially catabolized through the kynurenine pathway. In this study, tryptophan and kynurenine levels were increased, while alanine (a downstream product) was decreased, which suggested Dex mediated inhibition of kynureninase causing a backlog and accumulation of tryptophan and its hydroxylated product serotonin (5-HT) as shown in **Figure 4B**. Serotonin, a biogenic monoamine, is an essential neurotransmitter both in the central and peripheral nervous systems and following its release, it is actively cleared from synaptic clefts by SLC6A4. More recently, the emerging role of serotonin in bone metabolism and health had been investigated and clarified (Mohammad-Zadeh et al., 2008; Chabbi-Achengli et al., 2012; de Vernejoul et al., 2012; Yousri et al., 2017). Serotonin appears to have an inhibitory effect both on bone formation as well as resorption by reducing proliferation of murine primary osteoblasts (inhibiting nitric oxide release) and decreased osteoclastogenesis respectively (Yadav et al., 2008). In growing and mature tryptophan hydroxylase (Tph1^-/-^), serotonin deficient knockout mice, bone resorption was markedly reduced as evidenced by fewer osteoclasts, a phenotype that could be rescued by rescued by adding serotonin (Chabbi-Achengli et al., 2012). Liu et al. (2012) found the hydroxylation of tryptophan to serotonin was increased in the osteoporotic model, and tryptophan level was increased significantly, which reflects negatively on the catabolism of tryptophan to alanine (Liu et al., 2012).

The hepatic gluconeoneogenic enzyme tyrosine aminotransferase (TAT), as well as tyrosine hydroxylase, are glucocorticoid dependent enzymes which regulate tyrosine content in the blood which had been suggested to be an index of glucocorticoid action on metabolism (Williams et al., 1981; Rass, 2010). Also, under certain conditions, glucocorticoids can induce the expression of the hepatic phenylalanine hydroxylase enzyme (Bristeau et al., 2001). Therefore, the dysregulation of phenylalanine and tyrosine levels is probably the result of these complex effects of Dex on various GC responsive hepatic enzymes. Phenylalanine and tyrosine levels have been associated with osteoporosis as through altering Ca homeostasis by activating the Ca receptor, and reducing the level of PTH, which initiates the process of demineralization and bone resorption (Wu et al., 2015). The induction of phenylalanine hydroxylase and increase in the downstream metabolic expression such as the stress hormones (adrenaline and noradrenaline) activate the hormone-sensitive lipase (LIPE) via cyclic AMP-dependent protein kinase (PKA), which phosphorylates LIPE. The increasing activity of LIPE supports the increased level of some serum acylcarnitines.

As shown in **Figures 3A,D**, we observed overexpression of succinylacetone (4,6-dioxoheptanoic acid), a tyrosine metabolite which typically accumulates in patients with tyrosinemia type 1 who have recessive mutations in the hepatic enzyme fumarylacetoacetate hydrolase (FAH). This finding suggests a novel inhibitory effect for dexamethasone on FH. Therefore, dexamethasone and similar glucocorticoids should probably be used with caution in human patients with tyrosinemia type 1.

Finally, animal studies have shown that dietary supplements with certain amino acids, particularly L-lysine and arginine promote Ca absorption (Civitelli et al., 1992), and accordingly increase BMD, and serum OC. In postmenopausal women, administration of arginine-lysine-glycerophosphoric acid-lactose combination increased BMD and plasma osteocalcin, while serum PTH and hydroxyproline levels were reduced suggesting clinical benefit (Bellati and Liberati, 1994). These observations and the perturbed amino acids metabolism suggest a possible novel therapeutic interventional window to prevent or mitigate the effects of steroid-induced osteoporosis and related side effects. Further studies are necessary to validate these observations.

## Conclusion

Dexamethasone side effects are associated with several metabolic abnormalities, where LC-MS/MS-based metabolomic profiling of rats sera suggests a significant perturbation in several pathways involving mainly the metabolism of amino acid, pyrimidine, and nitrogen. Collectively, these disturbances strongly correlate with various known side effects such as reduced weight gain, hyperglycemia, dyslipidemia, and abnormal bone turnover found in our study. Significantly differentially expressed metabolites (hydroxyproline, tryptophan, alanine, phenylalanine, kynurenine, tryptophan, and tyrosine) identified in Dex-treated rats may serve as biomarkers able to predict the development of Dex related side effects. Longitudinal monitoring and evaluation of these biomarkers over the course of exposure to Dex in susceptible subjects will help determine their sensitivity and reliability in the diagnosis, prognosis, and therapeutic response. Expanded untargeted metabolomics and lipidomics profiling will also likely yield additional potentially useful biomarkers.

## Ethics Statement

This study was carried out in accordance with the recommendations of the Animal Care and Use Committee (ACUC). The protocol was approved by the “ACUC”.

## Author Contributions

AAR, KA, and AM have designed the experiment in help with FA and GM to establish the animal model at the animal facility. AM and MJ with the supervision of AAR and MD performed the metabolomics analysis. AA, GM, and FA have done the clinical evaluation and routine lab work. AAF has performed the radiological evaluation.

## Conflict of Interest Statement

The authors declare that the research was conducted in the absence of any commercial or financial relationships that could be construed as a potential conflict of interest.

## Footnotes

1 http://www.sample-size.net

2 http://www.metaboanalyst.ca

3 http://www.cytoscape.org

## Supplementary Material

FIGURE S1 Box plots and kernel density plots before and after normalization. The boxplots show ~50 features due to space limitations. The density plots are based on all samples. Selected methods: Row-wise normalization: Normalization to the constant sum; Data transformation: Log Normalization; Data scaling: Pareto Scaling.

TABLE S1 List of metabolites and their optimized LC/MS parameters. ^∗^Internal standards.

TABLE S2 Important features identified by volcano plot analysis.
