# Supplementary figures and images for: Metabolomics Based Profiling of Dexamethasone Side Effects in Rats
Source: Front Pharmacol. 2018 Feb 16;9:46. doi: 10.3389/fphar.2018.00046 (PMC5820529; doi:10.3389/fphar.2018.00046)

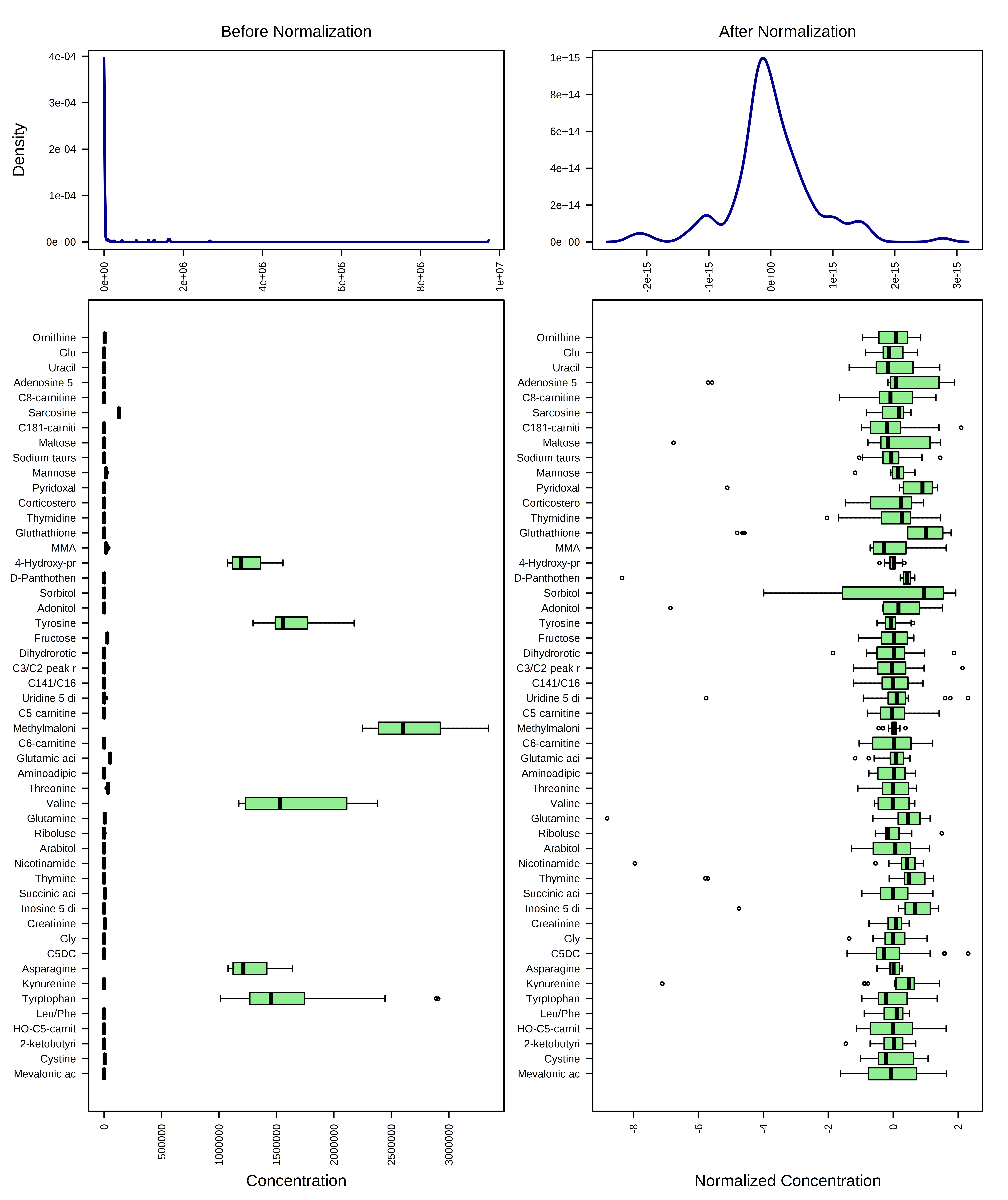

Supplement: FIGURE S1 — Box plots and kernel density plots before and after normalization. The boxplots show ~50 features due to space limitations. The density plots are based on all samples. Selected methods: Row-wise normalization: Normalization to the constant sum; Data transformation: Log Normalization; Data scaling: Pareto Scaling. [file Image_1.tif]
