# Supplementary material for: Metabolomics Based Profiling of Dexamethasone Side Effects in Rats
Source: Front Pharmacol. 2018 Feb 16;9:46. doi: 10.3389/fphar.2018.00046 (PMC5820529; doi:10.3389/fphar.2018.00046)
Supplement: TABLE S1 — List of metabolites and their optimized LC/MS parameters. ∗Internal standards. [file Table_1.docx]

| **Name** | **RT**  **(min)** | **Precursor ion (m/z)** | **Product ion (m/z)** | **Cone voltage**  **(V)** | **Collision energy**  **(CE)** | **HMDB** | **PubChem** | **KEGG** |
| --- | --- | --- | --- | --- | --- | --- | --- | --- |
| 17 α-Hydroxy pregnenolone | 8.49 | 333 | 133 | 25 | 22 | HMDB00363 | 91451 | C05138 |
| 17 α-Hydroxy progesterone | 8.73 | 331 | 97 | 65 | 20 | HMDB00374 | 6238 | C01176 |
| 2,3-Pyridine dicarboxylic (Quinolinic) acid | 4.09 | 166 | 122 | 20 | 11 | HMDB0000232 | 16219881 | C03722 |
| 21-Deoxycortisol | 5.8 | 345 | 87 | 25 | 18 | HMDB04030 | 222803 | C05497 |
| 2-hydroxyglutaric acid | 0.75 | 193 | 175 | 30 | 13 | HMDB06213 | 4394846 | C02917 |
| 2-Isopropyl malic acid | 4.13 | 175 | 115 | 32 | 16 | HMDB00402 | 5280523 | C02504 |
| 2-Ketobutyric acid | 4.28 | 101 | 57 | 52 | 8 | HMDB00005 | 58 | C00109 |
| 2-Phosphoglyceric acid | 5.2 | 187 | 131 | 25 | 18 | HMDB00362 | 59 | C00631 |
| 3,4-Dihydroxyphenyl acetic acid | 3.57 | 169 | 123 | 25 | 13 | HMDB0001336 | 547 | C01161 |
| 3-Hydroxyanthranilic acid | 3.51 | 152 | 91 | 32 | 22 | HMDB01476 | 86 | C00632 |
| 3-Methylglutaric acid | 4.16 | 145 | 83 | 28 | 14 | HMDB00752 | 12284 | NA |
| 3-Methylhistidine | 0.86 | 170 | 109 | 38 | 20 | HMDB00479 | 64969 | C01152 |
| 3-Methylmalonic acid | 4.29 | 117 | 73 | 30 | 12 | HMDB00202 | 487 | C02170 |
| 3-Phosphoglyceric acid | 4.4 | 185 | 79 |  |  | HMDB00807 | 724 | C00197 |
| 4,6-Dioxoheptanoic acid (succinylacetone) | 3.49 | 157 | 99 | 24 | 8 | HMDB0000635 | 5312 | NA |
| 4-Hydroxy-L-glutamic acid | 2.72 | 163 | 100 | 20 | 15 | HMDB02273 | 5460078 | C03079 |
| 4-Hydroxyphenyl pyruvic acid | 3.89 | 179 | 107 | 20 | 10 | HMDB00707 | 979 | C01179 |
| 4-Hydroxyproline | 2.01 | 132 | 69 | 2 | 16 | HMDB00725 | 5810 | C01157 |
| 5,6-Dihydro-5-methyl uracil | 1.62 | 127 | 42 | 17 | 15 | HMDB00079 | 93556 | C00906 |
| 5,6-Dihydrouridine | 1.57 | 277 | 194 | 24 | 10 | HMDB00497 | 94312 | C00429 |
| 5-Aminolevulinic acid | 1.1 | 130 | 112 | 30 | 12 | HMDB01149 | 137 | C00430 |
| 5-Hydroxyindoleacetic acid | 3.92 | 192 | 146 | 25 | 15 | HMDB00763 | 1826 | C05635 |
| 5-Hydroxylysine | 0.9 | 163 | 128 | 24 | 10 | HMDB00450 | 3032849 | C16741 |
| 6-Phosphogluconic acid | 4.23 | 275 | 97 | 68 | 19 | HMDB01316 | 91493 | C00345 |
| Acetyl-CoA | 5.61 | 808 | 408 | 20 | 40 | HMDB01206 | 444493 | C00024 |
| Adenine | 1.47 | 134 | 107 | 24 | 24 | HMDB00034 | 190 | C00147 |
| Adenosine | 2.34 | 265.95 | 133.9 | 32 | 22 | HMDB00050 | 60961 | C00212 |
| Adenosine diphosphate | 4.6 | 425.85 | 133.88 | 48 | 24 | HMDB01341 | 6022 | C00008 |
| Adenosine monophosphate | 3.57 | 345.87 | 96.89 | 42 | 24 | HMDB00045 | 6083 | C00020 |
| Adenosine triphosphate | 5.41 | 505.83 | 158.83 | 48 | 46 | HMDB00538 | 5957 | C00002 |
| Adenylosuccinic acid | 4.96 | 462 | 134 | 62 | 46 | HMDB00536 | 440122 | C03794 |
| Adonitol | 1.1 | 151 | 89 | 24 | 10 | HMDB00508 | 6912 | C00474 |
| Alanine | 0.94 | 90 | 72 | 20 | 6 | HMDB0000161 | 5950 | C01401 |
| Aldosterone | 4.86 | 359 | 199 | 15 | 17 | HMDB00037 | 24758425 | C01780 |
| Aminoadipic acid | 2.19 | 160 | 116 | 28 | 12 | HMDB00510 | 469 | C00956 |
| Arabinose | 1.03 | 149 | 89 | 25 | 7 | HMDB29942 | 66308 | C00259 |
| Argininosuccinic acid | 2.27 | 289 | 131 | 25 | 27 | HMDB00052 | 16950 | C03406 |
| Betaine | 1.28 | 118 | 59 | 36 | 16 | HMDB00043 | 247 | C00719 |
| Biopterin | 1.38 | 236 | 192 | 30 | 15 | HMDB00468 | 445040 | C06313 |
| Biotin | 4.46 | 245 | 227 | 50 | 25 | HMDB00030 | 171548 | C00120 |
| Cholic acid | 6.71 | 407 | 343 | 78 | 42 | HMDB00619 | 221493 | C00695 |
| Citrulline | 1.02 | 176 | 70 | 30 | 26 | HMDB00904 | 9750 | C00327 |
| Coenzyme A | 5.5 | 766 | 79 | 160 | 65 | HMDB01423 | 6816 | C00010 |
| Corticosterone | 7.69 | 347.05 | 121 | 48 | 24 | HMDB01547 | 5753 | C02140 |
| Cortisone | 6.82 | 361 | 163 | 55 | 25 | HMDB02802 | 222786 | C00762 |
| Creatinine | 1 | 114 | 44 | 20 | 13 | HMDB00562 | 588 | C00791 |
| Cyclic AMP | 3.54 | 327.8 | 133.9 | 46 | 22 | HMDB00058 | 6076 | C00575 |
| Cyclic GMP | 3.32 | 361.9 | 78.9 | 28 | 26 | HMDB01314 | 24316 | C00942 |
| Cysteine-S-sulfate | 2.72 | 200 | 81 | 20 | 16 | HMDB00731 | 115015 | C05824 |
| Cytidine | 1.1 | 241.9 | 108.9 | 32 | 10 | HMDB00089 | 6175 | C00475 |
| Cytidine mono phosphate-N-acetyl neuraminic acid (Sialic acid) | 2.36 | 637 | 346 | 30 | 25 | HMDB01176 | 448209 | C00128 |
| Cytidine monophosphate | 3.1 | 322.9 | 96.9 | 38 | 22 | HMDB00095 | 6131 | C00055 |
| Cytidine triphosphate | 5.28 | 481.8 | 158.83 | 46 | 30 | HMDB00082 | 6176 | C00063 |
| Cytosine | 1.73 | 112 | 85 | 13 | 12 | HMDB00630 | 597 | C00380 |
| Cytosine diphosphate | 4.17 | 401.83 | 158.84 | 44 | 26 | HMDB01546 | 6132 | C00112 |
| D-Arabitol | 1.02 | 151 | 89 | 30 | 10 | HMDB00568 | 827 | C01904 |
| D-Aspartic acid | 2.34 | 132 | 88 | 27 | 13 | HMDB06483 | 83887 | C00402 |
| Deoxyadenosine | 4.8 | 249.9 | 133.9 | 30 | 16 | HMDB00101 | 13730 | C00559 |
| Deoxyadenosine monophosphate | 3.42 | 329.8 | 194.9 | 100 | 16 | HMDB00905 | 12599 | C00360 |
| Deoxycytidine | 1.7 | 228 | 112 | 25 | 15 | HMDB00014 | 13711 | C00881 |
| Deoxycytosine monophosphate | 2.79 | 305.8 | 78.8 | 36 | 24 | HMDB01202 | 13945 | C00239 |
| Deoxyguanosine | 1.54 | 265.9 | 149.9 | 40 | 16 | HMDB00085 | 187790 | C00330 |
| Deoxyinosine | 1.5 | 250.82 | 134.87 | 42 | 20 | HMDB00071 | 65058 | C05512 |
| Deoxyribose-5’-phosphate | 2.83 | 212.89 | 96.83 | 26 | 16 | HMDB01031 | 45934311 | C00673 |
| Deoxyuridine | 1.33 | 227 | 94 | 24 | 10 | HMDB00012 | 13712 | C00526 |
| D-Erythrose-4-phosphate | 3.07 | 199 | 97 | 21 | 8 | HMDB01321 | 122357 | C00279 |
| Dexamethasone | 5.76 | 391 | 361 | 20 | 18 | HMDB15364 | 5743 | C15643 |
| Dextrose | 1.12 | 179 | 89 | 25 | 10 | HMDB00122 | 5793 | C00031 |
| D-Fructose | 0.93 | 179 | 89 | 16 | 8 | HMDB00660 | 439709 | C02336 |
| D-Galactono-1,4-lactone | 1.07 | 177 | 129 | 26 | 15 | HMDB0002541 | 6857365 | C01115 |
| D-Glucose | 0.95 | 179 | 89 | 25 | 10 | HMDB00122 | 5793 | C00031 |
| D-Glucuronic acid | 2.45 | 193 | 113 | 20 | 17 | HMDB00127 | 444791 | C00191 |
| D-Glutamic acid | 0.9 | 148 | 84 | 24 | 12 | HMDB03339 | 23327 | C00217 |
| Dihydrouracil | 1.01 | 113 | 69 | 26 | 58 | HMDB00076 | 649 | C00429 |
| Dihydroxyacetone phosphate | 4.5 | 169 | 79 | 25 | 17 | HMDB01473 | 668 | C0011 |
| Dimethylallylpyrophosphate (DMAPP) | 4.96 | 245 | 74 | 34 | 16 | HMDB01120 | 647 | C00235 |
| DL-Homocystine | 1.14 | 269 | 136 | 30 | 10 | HMDB00575 | 10010 | C01817 |
| D-Maltose | 1.02 | 341 | 101 | 30 | 20 | HMDB00163 | 10991489 | C00208 |
| D-Mannose | 0.95 | 179 | 59 | 25 | 17 | HMDB00169 | 18950 | C00159 |
| D-Mannose-1-phosphate | 2.73 | 259 | 79 | 25 | 20 | HMDB06330 | 644175 | C00636 |
| Dopamine | 1.06 | 154 | 91 | 26 | 19 | HMDB00073 | 681 | C03758 |
| D-Ribose-5-phosphate | 2.72 | 229 | 97 | 25 | 15 | HMDB01548 | 439167 | C00117 |
| D-Threitol | 2.51 | 123 | 105 | 30 | 8 | HMDB04136 | 169019 | C16884 |
| D-Tryptophan | 3.97 | 205 | 146 | 30 | 18 | HMDB13609 | 9060 | C00525 |
| Dulcitol | 1.15 | 181 | 101 | 50 | 15 | HMDB00107 | 11850 | C01697 |
| D-Xylitol | 1.04 | 153 | 117 | 22 | 8 | HMDB02917 | 6912 | C00379 |
| D-Xylose | 0.99 | 149 | 89 | 30 | 15 | HMDB00098 | 135191 | C00181 |
| Estradiol | 7.98 | 273 | 186 | 25 | 24 | HMDB00151 | 5757 | C00951 |
| Estriol | 4.64 | 287 | 145 | 25 | 40 | HMDB00153 | 5756 | C05141 |
| Estrone | 8.36 | 271 | 133 | 40 | 20 | HMDB00145 | 5870 | C00468 |
| Ethanolamine | 0.84 | 62 | 44 | 10 | 12 | HMDB00149 | 700 | C00189 |
| Fructose-1,6-bis phosphate | 4.56 | 339 | 97 | 34 | 19 | HMDB01058 | 172313 | C00354 |
| Fructose-6-phosphate | 2.69 | 259 | 197 | 30 | 15 | HMDB00124 | 69507 | C00085 |
| Fumaric acid | 6.1 | 115 | 71 | 30 | 6 | HMDB00134 | 444972 | C00122 |
| Galactose-1-phosphate | 2.81 | 259 | 79 | 25 | 20 | HMDB00645 | 123912 | C00446 |
| Gamma-Amino Butyric acid (GABA) | 0.98 | 104 | 69 | 26 | 14 | HMDB00112 | 119 | C00334 |
| Gluconolactone | 1.42 | 177 | 129 | 20 | 8 | HMDB00150 | 7027 | C00198 |
| Glucosamine-6-phosphate | 1.04 | 260 | 126 | 20 | 12 | HMDB01254 | 439217 | C00352 |
| Glucose-6-phosphate | 2.8 | 259 | 139 | 27 | 16 | HMDB01401 | 5958 | C00092 |
| Glutaric acid | 3.02 | 131 | 69 | 30 | 14 | HMDB00661 | 743 | C00489 |
| Glutathione | 1.9 | 308 | 179 | 18 | 12 | HMDB00125 | 124886 | C00051 |
| Glyceric acid | 1.33 | 251 | 145 | 25 | 18 | HMDB00139 | 439194 | C00258 |
| Glycerol | 1.05 | 93 | 57 | 20 | 8 | HMDB00131 | 753 | C00116 |
| Glycine | 0.95 | 76 | 30 | 19 | 12 | HMDB00123 | 750 | C00037 |
| Glycolic acid | 2.13 | 77 | 51 | 57 | 14 | HMDB00115 | 757 | C00160 |
| Glycoursodeoxycholic acid | 6.87 | 448 | 74 | 72 | 36 | HMDB00708 | 12310288 | NA |
| Guanine | 0.86 | 152 | 135 | 20 | 20 | HMDB00132 | 764 | C00242 |
| Guanosine | 1.5 | 281.85 | 149.8 | 42 | 18 | HMDB00133 | 6802 | C00387 |
| Guanosine diphosphate | 4.43 | 441.91 | 150 | 52 | 24 | HMDB01201 | 8977 | C00035 |
| Guanosine monophosphate | 4.8 | 362 | 79 | 28 | 29 | HMDB01397 | 6804 | C00144 |
| Guanosine triphosphate | 5.41 | 521.83 | 158.83 | 46 | 28 | HMDB01273 | 6830 | C00044 |
| Homocitrulline | 1.05 | 190 | 127 | 12 | 16 | HMDB00679 | 65072 | C02427 |
| Homo-L-arginine | 3.37 | 189 | 144 | 25 | 15 | HMDB00670 | 9085 | C01924 |
| Homovanillic acid | 4.34 | 183 | 94 | 18 | 26 | HMDB00118 | 1738 | C05582 |
| Hydroxykynurenine | 1.22 | 223 | 206 | 28 | 7 | HMDB00732 | 89 | C02794 |
| Hypoxanthine | 1.15 | 135 | 92 | 44 | 18 | HMDB00157 | 790 | C00262 |
| Indoleacetic acid | 5.86 | 176 | 103 | 34 | 30 | HMDB00197 | 802 | C00954 |
| Inosine | 1.41 | 266.88 | 134.93 | 44 | 26 | HMDB00195 | 6021 | C00294 |
| Inosine diphosphate | 4.39 | 426.9 | 134.92 | 50 | 22 | HMDB03335 | 644173 | C00104 |
| Inosine triphosphate | 5.41 | 507.38 | 441.9 | 62 | 60 | HMDB00189 | 8583 | C00081 |
| Isopentenyl pyrophosphate | 4.95 | 245 | 79 | 50 | 25 | HMDB01347 | 1195 | C00129 |
| Isoxanthopterin | 1.38 | 178 | 136 | 14 | 30 | HMDB00704 | 10729 | C03975 |
| Itaconic acid | 4.07 | 129 | 85 | 22 | 12 | HMDB02092 | 811 | C00490 |
| L-Acetylcarnitine | 1.26 | 204 | 85 | 20 | 21 | HMDB00201 | 7045767 | C02571 |
| L-Alloisoleucine | 2 | 132 | 69 | 20 | 18 | HMDB00557 | 99288 | C21096 |
| L-Arginine | 0.88 | 175 | 70 | 32 | 20 | HMDB00517 | 6322 | C00062 |
| L-Asparagine | 2.01 | 133 | 87 | 26 | 8 | HMDB00168 | 6267 | C00152 |
| L-Carnitine | 1.14 | 162 | 85 | 15 | 11 | HMDB00062 | 2724480 | C15025 |
| L-Cystathionine | 0.91 | 221 | 134 | 30 | 12 | HMDB00099 | 439258 | C02291 |
| L-Cystine | 3.37 | 241 | 109 | 32 | 20 | HMDB00192 | 67678 | C00491 |
| L-Dihydroorotic acid | 2.75 | 157 | 114 | 34 | 18 | HMDB02923 | 439216 | C00337 |
| L-Glutamine | 0.84 | 147 | 84 | 22 | 16 | HMDB00641 | 5961 | C00064 |
| L-Histidine | 0.86 | 83 | 83 | 26 | 22 | HMDB00177 | 6274 | C00135 |
| L-Homoserine | 1.1 | 118 | 100 | 30 | 8 | HMDB00719 | 12647 | C00263 |
| L-Isoleucine | 2.01 | 132 | 86 | 28 | 14 | HMDB00172 | 6306 | C00407 |
| Lithium acetoacetate | 1.97 | 109 | 65/51 | 30 | 13 | HMDB0000060 | 96 | C00164 |
| L-Kynurenine | 1.89 | 207 | 144 | 25 | 17 | HMDB00684 | 161166 | C00328 |
| L-Lactic acid | 2.73 | 89 | 43 | 30 | 12 | HMDB00190 | 107689 | C00186 |
| L-Leucine | 2 | 132 | 69 | 26 | 16 | HMDB00687 | 6106 | C00123 |
| L-Lysine | 0.88 | 147 | 84 | 26 | 26 | HMDB00182 | 5962 | C00047 |
| L-Methionine | 1.62 | 150 | 56 | 22 | 16 | HMDB00696 | 6137 | C00073 |
| L-Monapterin (neopterin) | 1.07 | 251.85 | 191.95 | 34 | 16 | HMDB0000877 | 440842 | NA |
| L-Phenylalanine | 2.85 | 166 | 103 | 24 | 28 | HMDB00159 | 6140 | C00079 |
| L-Proline | 0.9 | 116 | 71 | 22 | 20 | HMDB00162 | 145742 | C00148 |
| L-Ribulose | 1.03 | 149 | 89 | 20 | 7 | HMDB03371 | 439204 | C00310 |
| L-Serine | 0.95 | 104 | 74 | 27 | 11 | HMDB00187 | 5951 | C00065 |
| L-Sorbose | 0.98 | 178.81 | 89 | 72 | 8 | HMDB01266 | 441484 | C08356 |
| L-threonine | 0.98 | 120 | 74 | 24 | 16 | HMDB00167 | 6288 | C00188 |
| L-Tyrosine | 2.05 | 182 | 91 | 24 | 26 | HMDB00158 | 6057 | C00082 |
| L-Valine | 1.36 | 118 | 72 | 24 | 18 | HMDB00883 | 6287 | C00183 |
| Malic acid | 4 | 133 | 71 | 24 | 14 | HMDB00744 | 525 | C00711 |
| Malonic acid | 3.89 | 103 | 59 | 20 | 10 | HMDB00691 | 867 | C00383 |
| Mannitol | 0.93 | 181 | 89 | 30 | 12 | HMDB00765 | 6251 | C00392 |
| Melibiose | 0.91 | 341 | 179 | 56 | 12 | HMDB00048 | 440658 | C05402 |
| Mevalonic acid-5-phosphate | 5.27 | 227 | 97 | 26 | 16 | HMDB01343 | 439400 | NA |
| Myoinositol | 2.36 | 181 | 109 | 18 | 10 | HMDB00211 | 892 | C00137 |
| N1-Acetylspermine | 0.95 | 245 | 99 | 25 | 22 | HMDB01186 | 916 | C02567 |
| N-Acetyl-D-glucosamine | 0.95 | 220 | 119 | 28 | 10 | HMDB00215 | 439174 | C00140 |
| N-Acetyl-D-glucosamine-6-phosphate | 2.99 | 300 | 97 | 20 | 20 | HMDB01062 | 440996 | C00357 |
| N-Acetylmannosamine | 1.03 | 221 | 126 | 24 | 17 | HMDB01129 | 11096158 | C00645 |
| N-Acetylneuraminic acid (NANA, Sialic acid) | 2.63 | 308 | 87 | 22 | 8 | HMDB00230 | 445063 | C19910 |
| N-Acetylputrescine | 3.79 | 131 | 72 | 30 | 13 | HMDB02064 | 122356 | C02714 |
| NADH (Coenzyme I) | 4.48 | 664.05 | 78.9 | 64 | 62 | HMDB01487 | 439153 | C00004 |
| NADP (Coenzyme II) | 4.4 | 741.9 | 619.9 | 28 | 18 | HMDB00217 | 5886 | C00006 |
| Neopterin | 1.07 | 252 | 192 | 30 | 15 | HMDB00845 | 4455 | C05926 |
| Niacinamide | 2.39 | 124 | 81 | 6 | 20 | HMDB01406 | 936 | C00153 |
| Nicotinamide ribotide | 6.3 | 335 | 123 | 30 | 22 | HMDB00229 | 14180 | C00455 |
| Nicotinic acid | 6.29 | 122 | 78 | 25 | 15 | HMDB01488 | 938 | C00253 |
| Nicotinic acid mononucleotide | 6.26 | 336 | 125 | 42 | 22 | HMDB01132 | 53477721 | C01185 |
| O-phosphoethanol amine | 1.97 | 142 | 81 | 26 | 20 | HMDB00224 | 1015 | C00346 |
| Ornithine | 0.9 | 134 | 71 | 18 | 14 | HMDB00214 | 6262 | C00077 |
| Orotic acid | 3.02 | 155 | 111 | 2 | 14 | HMDB00226 | 967 | C00295 |
| Orotidine-5'-monophosphate | 4.41 | 367 | 79 | 14 | 14 | HMDB00218 | 160617 | C01103 |
| O-Succinyl-L-homoserine | 0.96 | 218 | 117 | 30 | 11 | NA | 439406 | C01118 |
| Oxalacetic acid | 1.2 | 133 | 23 | 170 | 20 | HMDB00223 | 970 | C00036 |
| Oxalic acid | 6.13 | 89 | 61 | 18 | 4 | HMDB02329 | 971 | C00209 |
| Oxidized glutathione | 2.44 | 611.02 | 305.99 | 52 | 26 | HMDB03337 | 975 | C00127 |
| Oxoglutaric acid | 0.81 | 147 | 84 | 25 | 15 | HMDB00208 | 51 | C00026 |
| Pantothenic acid | 3.07 | 239 | 128 | 30 | 20 | HMDB00210 | 988 | C00864 |
| Phenylpyruvic acid | 4.7 | 163 | 91 | 20 | 15 | HMDB00205 | 997 | C00166 |
| Phosphoenolpyruvic acid | 4.5 | 167 | 79 | 20 | 12 | HMDB00263 | 1005 | C00074 |
| Phosphoserine | 5.9 | 186 | 88 | 44 | 26 | HMDB00272 | 68841 | C01005 |
| Pipecolic acid | 1.77 | 130 | 84 | 10 | 10 | HMDB00070 | 849 | C00408 |
| Porphobilinogen | 2.3 | 226 | 26 | 170 | 52 | HMDB00245 | 1021 | C00931 |
| Progesterone | 9.63 | 315 | 109 | 44 | 30 | HMDB01830 | 5994 | C00410 |
| Pterin | 1.38 | 162 | 119 | 30 | 15 | HMDB00802 | 73000 | C00715 |
| Putrescine | 0.73 | 89 | 72 | 45 | 9 | HMDB01414 | 1045 | C00134 |
| Pyridoxal Hydrochloride | 4.04 | 203 | 167 | 20 | 15 | HMDB0000239 | 6171 | C00314 |
| Pyridoxal-5'-phosphate | 4 | 246 | 97 | 27 | 10 | HMDB01491 | 1051 | C00018 |
| Raffinose | 1.07 | 505 | 163 | 25 | 20 | HMDB03213 | 10542 | C00492 |
| Ribitol | 0.96 | 151 | 89 | 24 | 10 | HMDB00508 | NA | C00474 |
| Saccharopine | 1.07 | 277 | 84 | 111 | 20 | HMDB00279 | 160556 | C00762 |
| Sarcosine | 0.97 | 90 | 62.8 | 26 | 6 | HMDB00271 | 1088 | C00213 |
| Sedoheptulose-7-phosphate | 2.72 | 289 | 97 | 50 | 12 | HMDB0001068 | 165007 | C05382 |
| Sepiapterin | 2.35 | 235.91 | 163.99 | 44 | 18 | HMDB00238 | 65253 | C00835 |
| Sodium 4-methyl-2-oxovalerate | 0.89 | 153 | 23 | 27 | 7 | HMDB0000695 | 70 | NA |
| Sodium glycodeoxycholate | 10.11 | 472.2 | 397 | 30 | 30 | HMDB32596 | 755 | C05464 |
| Sodium β-hydroxy isobutyrate | 5.39 | 127 | 84 | 30 | 12 | HMDB0000336 | 87 | C01188 |
| Sorbitol | 1.02 | 181 | 101 | 32 | 10 | HMDB00247 | 5780 | C00794 |
| Spermine | 0.73 | 203 | 129 | 20 | 18 | HMDB01256 | 1103 | C00750 |
| Sphingosine | 9.58 | 299 | 252 | 12 | 15 | HMDB00252 | 5353955 | C00319 |
| S-Sulfocysteine | 2.78 | 200 | 81 | 25 | 16 | HMDB00731 | 115015 | C05824 |
| Succinic acid | 4.05 | 117 | 73 | 30 | 15 | HMDB00254 | 1110 | C00042 |
| Taurine | 0.95 | 124 | 80 | 30 | 18 | HMDB00251 | 1123 | C00245 |
| Taurodeoxycholic acid | 9.48 | 522 | 147 | 46 | 28 | HMDB00896 | 2733768 | C05463 |
| Tauroursodeoxycholic Acid | 9.89 | 522.21 | 147.9 | 46 | 28 | HMDB00874 | 12443252 | C16868 |
| Thiamine | 3.16 | 266 | 77 | 40 | 18 | HMDB00235 | 1130 | C00378 |
| Thymidine | 2.1 | 241 | 42 | 30 | 20 | HMDB00273 | 5789 | C00214 |
| Thymine | 1.46 | 125 | 42 | 26 | 14 | HMDB00262 | 1135 | C00178 |
| Tryptophanol | 1.3 | 189 | 129 | 30 | 15 | HMDB03447 | 10685 | C00955 |
| Uracil | 3.01 | 111 | 42 | 30 | 15 | HMDB00300 | 1174 | C00106 |
| Ureidopropionic acid | 2.53 | 131 | 88 | 18 | 16 | HMDB00026 | 111 | C02642 |
| Uridine | 1.28 | 242.9 | 110.02 | 36 | 14 | HMDB00296 | 6029 | C00299 |
| Uridine diphosphate-N-acetylglucosamine | 4.23 | 606 | 282 | 30 | 28 | HMDB00290 | 445675 | C00043 |
| Uridine-5′-diphospho galactose | 2.18 | 611 | 449 | 100 | 20 | HMDB42037 | 18058 | NA |
| Uridine-5'-diphosphate | 4.39 | 402.8 | 158.8 | 44 | 28 | HMDB00295 | 6031 | C00015 |
| Uridine-5'-mono phosphate | 3.1 | 322.89 | 96.83 | 40 | 22 | HMDB00288 | 6030 | C00105 |
| Uridine-diphospo- glucuronic acid | 5.34 | 579 | 403 | 170 | 25 | HMDB00935 | 17473 | C00167 |
| Xanthine | 1.23 | 150.75 | 79.87 | 40 | 20 | HMDB00292 | 1188 | C00385 |
| β-Nicotinamide adenine dinucleotide (NAD) | 2.83 | 661.95 | 539.91 | 26 | 16 | HMDB0000902 | 5893 | C00003 |
| *2-Amino-1,6-Hexandioc acid-D3 | 2.33 | 163 | 144 | 30 | 16 | NA | NA | NA |
| *2-Deoxyadenosine-C13 | 2.6 | 271 | 154 | 150 | 15 | NA | NA | NA |
| *Adenosine C13 | 2.31 | 266.9 | 133.9 | 14 | 14 | NA | NA | NA |
| *Alanine-d1 | 0.98 | 91 | 45 | 28 | 10 | NA | 16213420 | NA |
| *Alanine-d4 | 0.95 | 94 | 78 | 18 | 6 | NA | 12205373 | NA |
| *Arginine-d7 | 0.91 | 182 | 77 | 32 | 20 | NA | 117065486 | NA |
| *Aspartate-d3 | 1.03 | 137 | 77 | 58 | 18 | NA | NA | NA |
| *C10-Carnitine-d3 | 8.39 | 319 | 85 | 18 | 22 | NA | NA | NA |
| *C12-Carnitine-d3 | 9.71 | 247 | 85 | 18 | 20 | NA | NA | NA |
| *C14-Carnitine-d3 | 10.89 | 375 | 85 | 18 | 24 | NA | NA | NA |
| *C16-Carnitine-d3 | 10.9 | 403 | 85 | 18 | 22 | NA | NA | NA |
| *C18-Carnitine-d3 | 10.18 | 431 | 85 | 52 | 24 | NA | NA | NA |
| *C2-Carnitine-d3 | 1.26 | 207 | 85 | 28 | 18 | NA | NA | NA |
| *C3-Carnitine-d3 | 2.46 | 221 | 85 | 30 | 18 | NA | NA | NA |
| *C4-Carnitine-d3 | 3.1 | 235 | 85 | 28 | 20 | NA | NA | NA |
| *C5-Carnitine-d9 | 3.86 | 255 | 85 | 28 | 18 | NA | NA | NA |
| *C6-Carnitine-d3 | 4.93 | 263 | 85 | 34 | 20 | NA | NA | NA |
| *C8-Carnitine-d3 | 6.8 | 291 | 85 | 16 | 20 | NA | NA | NA |
| *Carnitine-d9 | 2.78 | 171 | 106 | 24 | 24 | NA | 129880693 | NA |
| *Citric acid-d4 | 4.51 | 195 | 132 | 24 | 12 | NA | 16213286 | NA |
| *Citrulline-d2 | 1.03 | 178 | 72 | 22 | 20 | NA | 71309912 | NA |
| *D-Fructose(2-13C) | 0.99 | 180 | 90 | 25 | 10 | NA | NA | NA |
| *Glucose-d7 | 1.04 | 186 | 124 | 50 | 15 | NA | 71309927 | NA |
| *Glutamate-d5 | 0.99 | 153 | 88 | 22 | 14 | NA | 12248503 | NA |
| *Guanosine-15N5 | 1.48 | 155 | 137 | 50 | 15 | NA | NA | NA |
| *Inosine-15N4 | 1.35 | 271 | 139 | 30 | 25 | NA | NA | NA |
| *L-Citrulline-d7 | 1.04 | 181 | 138.13 | 30 | 15 | NA | 129850295 | NA |
| *Leucine-d3 | 1.92 | 135 | 89 | 22 | 24 | NA | 11073472 | NA |
| *Methionine-d3 | 0.99 | 153 | 88 | 22 | 16 | NA | 15556503 | NA |
| *Methyl malonate-d3 | 1.07 | 120 | 76 | 30 | 8 | NA | NA | NA |
| *Ornithine-d6 | 0.83 | 139 | 76 | 18 | 16 | NA | 71309858 | NA |
| *Phenylalanine-d5 | 2.82 | 171 | 103 | 22 | 26 | NA | 13000995 | NA |
| *Tyrosine-d4 | 5.33 | 186 | 57 | 76 | 24 | NA | 56613523 | NA |
| *Valine-d8 | 1.07 | 126 | 62 | 14 | 20 | NA | 90969676 | NA |

*Internal standards
