# Supplementary material for: Metabolomics Based Profiling of Dexamethasone Side Effects in Rats
Source: Front Pharmacol. 2018 Feb 16;9:46. doi: 10.3389/fphar.2018.00046 (PMC5820529; doi:10.3389/fphar.2018.00046)
Supplement: TABLE S2 — Important features identified by volcano plot analysis. [file Table_2.docx]

Table S2: Important features identified by volcano plot analysis

| **Name** | **FC** | **log_2_(FC)** | **p.value** |
| --- | --- | --- | --- |
| Glutamine | 6.3911 | 2.6761 | 2.06E-08 |
| Lysine | 6.8286 | 2.7716 | 1.14E-07 |
| Cysteine | 5.7641 | 2.5271 | 1.20E-07 |
| Alanine | 7.3314 | 2.8741 | 1.46E-07 |
| Tyrosine | 6.5717 | 2.7163 | 7.89E-07 |
| Leucine | 7.4857 | 2.9041 | 9.11E-07 |
| Citrulline | 6.2529 | 2.6445 | 1.93E-06 |
| Glycine | 7.0261 | 2.8127 | 2.59E-06 |
| Methionine | 6.9139 | 2.7895 | 4.20E-06 |
| Valine | 7.4341 | 2.8942 | 5.41E-06 |
| Isoleucine | 7.3042 | 2.8687 | 5.88E-06 |
| Phenylalanine | 5.9238 | 2.5665 | 9.05E-06 |
| Proline | 6.1611 | 2.6232 | 5.39E-05 |
| C4-carnitine | 0.41776 | -1.2592 | 0.000166 |
| Gama Amino Butyric acid | 0.48666 | -1.039 | 0.000737 |
| 4,6,Dioxoheptanoic acid | 0.032375 | -4.949 | 0.001279 |
| 1-Methylhistidine | 5.6231 | 2.4914 | 0.001693 |
| N-Acetylneuraminic acid | 0.49445 | -1.0161 | 0.002029 |
| Tyrptophan | 0.48001 | -1.0589 | 0.004175 |
| HO-C5-carnitine | 0.46813 | -1.095 | 0.005445 |
| Ornithine | 5.9047 | 2.5619 | 0.005615 |
| 5-Aminoleuvillinic acid | 3.6049 | 1.8499 | 0.005679 |
| Isoprophymallic acid | 0.38405 | -1.3806 | 0.006214 |
| C18:1-carnitine | 0.37129 | -1.4294 | 0.012234 |
| Sorbitol | 0.13312 | -2.9092 | 0.021589 |
| Arginine | 6.6993 | 2.744 | 0.022738 |
| CDP | 0.000332 | -11.558 | 0.03336 |
| 3-ureidopropionic acid | 0.38456 | -1.3787 | 0.036197 |
| C16-carnitine | 0.33116 | -1.5944 | 0.045795 |
| Mevalonic acid 5-phosphate | 0.45014 | -1.1516 | 0.047412 |
| C5DC-carnitine | 0.3444 | -1.5378 | 0.049422 |
| cAMP | 3244 | 11.664 | 0.055876 |
| AMP | 3526.4 | 11.784 | 0.056485 |
| Pyridoxal | 0.20054 | -2.318 | 0.066432 |
| 6-phosphogluconic acid | 0.44708 | -1.1614 | 0.079879 |
| IDP | 2.7123 | 1.4395 | 0.12445 |
| MMA | 0.49518 | -1.014 | 0.13363 |
| Adonitol | 4.9989 | 2.3216 | 0.13933 |
| Hydroxyproline | 0.079075 | -3.6606 | 0.14151 |
| Gluthathione | 6.5275 | 2.7065 | 0.18467 |
| Homovanillic acid | 0.24707 | -2.017 | 0.20478 |
| Glutamine | 0.38492 | -1.3774 | 0.23306 |
| Nicotinamide | 0.3968 | -1.3335 | 0.25556 |
| Dimethylallylpyrophoshate | 2.9148 | 1.5434 | 0.25994 |
| CMP | 3.1463 | 1.6537 | 0.28022 |
| Homocysteine | 0.45687 | -1.1301 | 0.29648 |
| GTP | 2.1072 | 1.0753 | 0.29719 |
| Cystathionine | 0.47954 | -1.0603 | 0.3025 |
| ADP | 3.6217 | 1.8566 | 0.32808 |
| UMP | 0.25622 | -1.9645 | 0.37751 |

| **Name** | **FC** | **log_2_(FC)** | **p.value** |
| --- | --- | --- | --- |
| AMP | 3526.4 | 11.784 | 0.056485 |
| cAMP | 3244 | 11.664 | 0.055876 |
| Leucine | 7.4857 | 2.9041 | 9.11E-07 |
| Valine | 7.4341 | 2.8942 | 5.41E-06 |
| Alanine | 7.3314 | 2.8741 | 1.46E-07 |
| Isoleucine | 7.3042 | 2.8687 | 5.88E-06 |
| Glycine | 7.0261 | 2.8127 | 2.59E-06 |
| Methionine | 6.9139 | 2.7895 | 4.20E-06 |
| Lysine | 6.8286 | 2.7716 | 1.14E-07 |
| Arginine | 6.6993 | 2.744 | 0.022738 |
| Tyrosine | 6.5717 | 2.7163 | 7.89E-07 |
| Gluthathione | 6.5275 | 2.7065 | 0.18467 |
| Glutamine | 6.3911 | 2.6761 | 2.06E-08 |
| Citrulline | 6.2529 | 2.6445 | 1.93E-06 |
| Proline | 6.1611 | 2.6232 | 5.39E-05 |
| Phenylalanine | 5.9238 | 2.5665 | 9.05E-06 |
| Ornithine | 5.9047 | 2.5619 | 0.005615 |
| Cysteine | 5.7641 | 2.5271 | 1.20E-07 |
| 1-Methylhistidine | 5.6231 | 2.4914 | 0.001693 |
| Adonitol | 4.9989 | 2.3216 | 0.13933 |
| ADP | 3.6217 | 1.8566 | 0.32808 |
| 5-Aminoleuvillinic acid | 3.6049 | 1.8499 | 0.005679 |
| CMP | 3.1463 | 1.6537 | 0.28022 |
| Dimethylallylpyrophoshate | 2.9148 | 1.5434 | 0.25994 |
| IDP | 2.7123 | 1.4395 | 0.12445 |
| GTP | 2.1072 | 1.0753 | 0.29719 |
| MMA | 0.49518 | -1.014 | 0.13363 |
| N-Acetylneuraminic acid | 0.49445 | -1.0161 | 0.002029 |
| Gama Amino Butyric acid | 0.48666 | -1.039 | 0.000737 |
| Tyrptophan | 0.48001 | -1.0589 | 0.004175 |
| Cystathionine | 0.47954 | -1.0603 | 0.3025 |
| HO-C5-carnitine | 0.46813 | -1.095 | 0.005445 |
| Homocysteine | 0.45687 | -1.1301 | 0.29648 |
| Mevalonic acid 5-phosphate | 0.45014 | -1.1516 | 0.047412 |
| 6-phosphogluconic acid | 0.44708 | -1.1614 | 0.079879 |
| C4-carnitine | 0.41776 | -1.2592 | 0.000166 |
| Niacinamide | 0.3968 | -1.3335 | 0.25556 |
| Glutamine | 0.38492 | -1.3774 | 0.23306 |
| 3-ureidopropionic acid | 0.38456 | -1.3787 | 0.036197 |
| Isoprophymallic acid | 0.38405 | -1.3806 | 0.006214 |
| C18:1-carnitine | 0.37129 | -1.4294 | 0.012234 |
| C5DC-carnitine | 0.3444 | -1.5378 | 0.049422 |
| C16-carnitine | 0.33116 | -1.5944 | 0.045795 |
| UMP | 0.25622 | -1.9645 | 0.37751 |
| Homovanillic acid | 0.24707 | -2.017 | 0.20478 |
| Pyridoxal | 0.20054 | -2.318 | 0.066432 |
| Sorbitol | 0.13312 | -2.9092 | 0.021589 |
| Hydroxyproline | 0.079075 | -3.6606 | 0.14151 |
| 4,6,Dioxoheptanoic acid | 0.032375 | -4.949 | 0.001279 |
| CDP | 0.000332 | -11.558 | 0.03336 |

| **Pathway** | **Total** | **Hits** | **Raw p** | **-Log(P)** | **Impact** |
| --- | --- | --- | --- | --- | --- |
| Aminoacyl-tRNA biosynthesis | 67 | 10 | 3.08E-07 | 14.993 | 0 |
| Arginine and proline metabolism | 44 | 6 | 0.000187 | 8.5867 | 0.31604 |
| Pyrimidine metabolism | 41 | 4 | 0.008808 | 4.7321 | 0.08466 |
| Alanine, aspartate and glutamate metabolism | 24 | 3 | 0.012032 | 4.4202 | 0.26371 |
| Nitrogen metabolism | 9 | 2 | 0.013598 | 4.2979 | 0 |
| Valine, leucine and isoleucine biosynthesis | 11 | 2 | 0.020248 | 3.8997 | 0.66666 |
| Phenylalanine, tyrosine and tryptophan biosynthesis | 4 | 1 | 0.08029 | 2.5221 | 0.5 |
| Glutathione metabolism | 26 | 2 | 0.098895 | 2.3137 | 0.00573 |
| Galactose metabolism | 26 | 2 | 0.098895 | 2.3137 | 0.03644 |
| D-Glutamine and D-glutamate metabolism | 5 | 1 | 0.099369 | 2.3089 | 0 |
| Biotin metabolism | 5 | 1 | 0.099369 | 2.3089 | 0 |
| Cysteine and methionine metabolism | 28 | 2 | 0.11217 | 2.1878 | 0.09464 |
| Cyanoamino acid metabolism | 6 | 1 | 0.11806 | 2.1365 | 0 |
| Glycine, serine and threonine metabolism | 32 | 2 | 0.14 | 1.9661 | 0.29197 |
| Taurine and hypotaurine metabolism | 8 | 1 | 0.15434 | 1.8686 | 0.42857 |
| Purine metabolism | 68 | 3 | 0.16275 | 1.8156 | 0.05757 |

Table S2: Detailed pathway analysis of significantly differentially expressed metabolites
